# Serum Zinc-*α*2-Glycoprotein Levels in Patients with or without Coronary Artery Disease in Chinese North Population

**DOI:** 10.1155/2020/7864721

**Published:** 2020-02-27

**Authors:** Meijuan Liu, Zeyu Liu, Huijuan Zhu, Linjie Wang, Hongbo Yang, Kemin Yan, Fengying Gong, Hui Pan, Yong Zeng

**Affiliations:** ^1^Key Laboratory of Endocrinology of National Health Commission, Department of Endocrinology, Peking Union Medical College Hospital, Chinese Academy of Medical Science and Peking Union Medical College, Beijing 100730, China; ^2^Department of Cardiology, Beijing Anzhen Hospital, Capital Medical University, Beijing, China

## Abstract

Coronary artery disease (CAD), the leading cause of morbidity and mortality, has imposed huge health and economic burdens globally. Zinc-*α*2-glycoprotein (ZAG) is a novel adipokine. Increasing evidence suggests the close relationship between serum ZAG levels and various cardiometabolic risk factors. However, the relationship between serum ZAG levels and CAD is still not fully clarified. We conducted this study to evaluate serum ZAG levels and its association with cardiovascular risk factors. A total of 129 patients with CAD, 99 patients with noncoronary artery disease (NCAD), and 121 controls were recruited in this retrospective study. CAD (coronary artery stenosis ≥50%) or NCAD (coronary artery stenosis <50%) patients who underwent coronary angiography were diagnosed according to the American Heart Association criteria. Serum ZAG levels were determined via commercial enzyme-linked immunosorbent assay (ELISA) kits. The results showed that serum ZAG levels in CAD and NCAD groups were significantly decreased when compared with those in the control group. Multiple stepwise regression analysis revealed that the grouping variable (control, NCAD, and CAD) was an independent determinant of serum ZAG levels (*β* = −0.328, *P* < 0.001) after controlling other confounding factors. Further multivariate ordinary logistic regression analysis demonstrated that the risk of grouping at one level higher in subjects with the lowest tertile of ZAG levels was 2.28-fold higher than those with the highest tertile levels (OR = 3.281, 95% CI 1.782–6.038, *P* < 0.001). The receiver-operating characteristic (ROC) curve analysis showed that serum ZAG could distinguish CAD patients (AUC = 0.706, 95% CI, 0.643–0.770, *P* < 0.05), NCAD patients (AUC = 0.673, 95% CI, 0.602–0.743, *P* < 0.05), and NCAD and CAD patients (AUC = 0.692, 95% CI, 0.633–0.750, *P* < 0.05) from controls. In conclusion, serum ZAG levels were significantly decreased in NCAD/CAD patients. The decreased serum ZAG levels were independently associated with the presence of NCAD/CAD. ZAG might serve as a candidate diagnostic biomarker for NCAD/CAD.

## 1. Introduction

Cardiovascular disease is an alarming healthcare concern and has become the leading cause of morbidity and mortality globally [1, 2]. According to the data provided by the Global Burden of Disease Study 2016 (GBD 2016), approximately 17.6 million deaths worldwide in 2016 were caused by cardiovascular disease, and among them, coronary artery disease (CAD) accounted for 30% [3]. Although numerous countermeasures have been taken, CAD still caused huge economic pressure on government and patients [4]. In this scenario, actions that are helpful for the early detection and meaningful intervention of CAD have caught the attention of international leaders [5, 6]. Dyslipidemia, hypertension, smoking, and type 2 diabetes mellitus (T2DM) are well-known key risk factors for CAD [1]; currently, numerous studies have pointed out that obesity is also an independent risk factor for CAD [7–10]. Based on the national survey data of children and adolescents performed in the US from 1963 to 2002, a 10 kg increase in body weight causes a 12% rise in the risk of CAD [7].

Far from being a passive energy reservoir pool, adipose tissue acts as a highly active endocrine organ by producing numerous biological activity factors, collectively named as adipokines [11–13]. Although the pathophysiology of the close relationship between obesity and CAD is complex and remains incompletely understood, the dysregulation of the adipokines in obesity has been reported to play a crucial role in the development of CAD [12–16]. For instance, adiponectin, the most extensively studied adipokine, has various beneficial effects in the cardiovascular system through its anti‐inflammatory and antioxidative properties [12]. Leptin exerts multiple detrimental cardiovascular roles such as promoting angiogenesis and arterial thrombosis, stimulating immunological responses and inflammatory reaction, and impairing arterial distensibility [17]. Resistin also has detrimental cardiovascular effects through stimulating oxidative stress response and aggravating ischemia/reperfusion injury [12]. Serum resistin levels have been reported to serve as independent predictors of future fatal cardiovascular events [18].

Zinc-a2-glycoprotein (ZAG) has been identified as a novel adipokine, which is not only highly expressed in the subcutaneous and visceral white adipose tissue of mice and humans but also could be secreted by human adipocytes [19]. ZAG has been demonstrated to inhibit lipogenesis and promote the lipolysis and *β*-oxidation of fatty acids [20]. Additionally, ZAG has been reported to promote glucose utilization and to regulate insulin sensitivity [20]. Previous studies have shown the close relationship between serum ZAG levels and various cardiometabolic risk factors, such as obesity [21, 22], T2DM [23, 24], hypertension [25], cigarette smoking [26], and metabolic syndrome [27, 28]. In a recent study performed by Smékal et al. in 65 Caucasians, decreased plasma ZAG levels were observed in premature CAD (PCAD) patients and plasma ZAG levels might serve as potential biomarkers for the diagnosis of PCAD with the area under the curve (AUC) of the receiver operating characteristic curve (ROC) was 0.89 [14]. PCAD refers to the diagnosis with CAD in males before the age of 55 or females before the age of 65 [29]. Our recently published data in Chinese population also demonstrated the significantly decreased serum ZAG levels in PCAD patients [30]. In studies to date, the relationship between serum ZAG levels and CAD was performed in middle-aged populations. Aging was found to be one of the major risk factors for CAD, and CAD mainly occurred in the old patients. It is significant and necessary to investigate serum ZAG levels in CAD patients, especially in the elderly population.

Therefore, the purposes of this study were (1) to determine serum ZAG in 129 CAD patients, 99 noncoronary artery disease (NCAD) patients, and 121 controls; (2) to explore the associations between serum ZAG and CAD‐related risk variables; and (3) to compare the diagnostic power of serum ZAG for discriminating CAD patients from controls.

## 2. Methods

### 2.1. Study Population

Between November 2011 and April 2016, patients who underwent coronary angiography at the Department of Cardiology in Peking Union Medical College Hospital (PUMCH) due to typical chest pain or chest congestion or positive noninvasive test results (i.e., electrocardiogram suggestive of ischemia, suspicious myocardial perfusion scan, or positive exercise tolerance test) were retrospectively studied in the present study. The coronary angiography was evaluated by an experienced cardiologist at the cardiac catheterization laboratory using digital subtraction cardiovascular contrast machine and a quantitative coronary angiographic system. The inclusion criterion for CAD was symptomatic patients with any coronary artery stenosis ≥50% [5] and for NCAD was symptomatic patients with all coronary arteries stenosis <50% [5]. The exclusion criteria were the history of coronary artery stent or bypass graft, the finding of myocardial bridge during angiography, incomplete information, and any diseases that may interfere with results, such as active period of autoimmune disease, infectious diseases, severe liver or kidney dysfunction, aortic dissection, and aneurysm. A total of 228 patients were finally enrolled in our present study, with 129 subjects in the CAD group and 99 subjects in the NCAD group. In the NCAD group, the proportions of patients with hypertension, T2DM, and hyperlipidemia were 65.66%, 28.28%, and 53.54%, respectively. In the CAD group, the proportions of patients with hypertension, T2DM, and hyperlipidemia were 72.87%, 50.39%, and 65.12%, respectively. Significantly, more patients in the CAD group were treated with hypoglycemic drugs than patients in the NCAD group (31.01% vs. 18.18%, *P* < 0.05). Additionally, 121 subjects with normal hepatic and renal function, normal blood and urine routine analysis, and no history of diabetes, hypertension, and heart disease were chosen from the physical examination center in PUMCH to serve as the control group. Written informed consent was signed by each participant, and the present study was approved by the ethics committee of PUMCH (No. S-K205). All methods were carried out in accordance with the relevant guidelines and regulations.

### 2.2. Anthropometric Evaluation

Body weight and height of all subjects were measured, and the body mass index (BMI) was determined. The minimum measurement sensitivity of body height and body weight was 0.1 cm and 0.1 kg, respectively. BMI was calculated by the following formula: body weight (kg)/height squared (m^2^). Using mercury sphygmomanometer, blood pressure was measured twice with all subjects in sitting positions. The average of the two measurements was calculated and recorded.

### 2.3. Blood Samples Collection and Biochemical Variable Examination

Following an overnight fast, blood samples were taken from all subjects and then separated by using a centrifuge. The centrifuged serum samples were put into a 1.5 mL Eppendorf tube and stored at −80°C. The fasting blood glucose (FBG) concentration, total cholesterol (TC), triglycerides (TG), high-density lipoprotein cholesterol (HDL-C), low-density lipoprotein cholesterol (LDL-C), alanine transaminase (ALT), creatinine (Cr), and urea were determined via conventional automated laboratory methods in the clinical laboratory of PUMCH.

### 2.4. Measurements of ZAG Levels

Serum ZAG concentrations were measured using commercially available human enzyme-linked immunosorbent assay (ELISA) kits according to the instructions of the manufacturer (Catalogue No. SEL231Hu, USCN Life Science Inc., Wuhan, China). The detection range for ZAG was 4.7–300 ng/mL. The intra-assay coefficient of variation (CV) for ZAG was 5.64%. The inter-assay CV for ZAG was 12.82%.

### 2.5. Sample Size and Statistical Analysis

The required sample size was calculated by using the MedSci Sample Size tools (MSST). Based on our previous studies that were conducted on the PCAD patients, the mean values of serum ZAG levels in the PCAD, nonpremature CAD (NPCAD), and control groups were 8.03, 8.28, and 8.78, respectively; the standard deviation (SD) in the PCAD, NPCAD, and control groups were 1.01, 1.61, and 1.89, respectively [30]. Assuming a two-sided type I error (a) of 0.05 and a power of 0.80, and equal sample sizes in the two groups, 70 patients were required.

Data were represented as mean ± SD or median with interquartile range. The Shapiro–Wilk test was used to evaluate the normal distribution of the variables in each group. The chi-square test was used for the comparison of categorical data. The independent sample *t*-test or Mann–Whitney *U* test was performed to compare variables between two groups. And the one-way ANOVA test was performed for the comparison of variables among three groups. To determine the relationship between serum ZAG and other variables, partial correlation analysis was used. To identify independent factors associated with serum ZAG, multiple stepwise regression analysis was conducted. Moreover, the link between serum ZAG levels (tertile) and CAD risks was explored via multivariate ordinary logistic regression analysis. Finally, the AUC and the sensitivity and specificity of serum ZAG levels in distinguishing CAD patients from controls were estimated by the ROC analysis. All statistical computations were performed using SPSS 20.0 for Windows (SPSS Inc., Chicago, IL, USA). A *P* value of <0.05 was considered statistically significant.

## 3. Results

### 3.1. Baseline Characteristics of Subjects in CAD, NCAD, and Control Groups

Baseline characteristics of subjects in CAD, NCAD, and control groups were presented in Table 1. As expected, compared to the controls, patients in CAD and NCAD groups exhibited higher FBG, ALT, TC/HDL-C and lower HDL-C levels (*P* all <0.05). In the CAD group, systolic blood pressure (SBP), LDL-C/HDL-C, and urea levels were higher than those in the control group, and FBG, LDL-C/HDL-C, and the percentage of T2DM were higher than those in the NCAD group (*P* all <0.05). However, the diastolic blood pressure (DBP) in the CAD group and the TC and LDL-C in CAD and NCAD groups were significantly lower than those in the control group (*P* all <0.05) probably due to the use of hypotensive and lipid‐lowering drugs. Additionally, other variables, including TG and Cr, showed no significant difference among the three groups. Significantly more patients in the CAD group were treated with hypoglycemic drugs than patients in the NCAD group (31.01% vs. 18.18%, *P* < 0.05). No significant difference was found with respect to BMI among the three groups. Compared with the control group, the CAD and NCAD groups were older and exhibited a higher proportion of males (*P*<0.05).

### 3.2. Serum ZAG Levels in CAD, NCAD, and Control Groups

**Figure 1:**
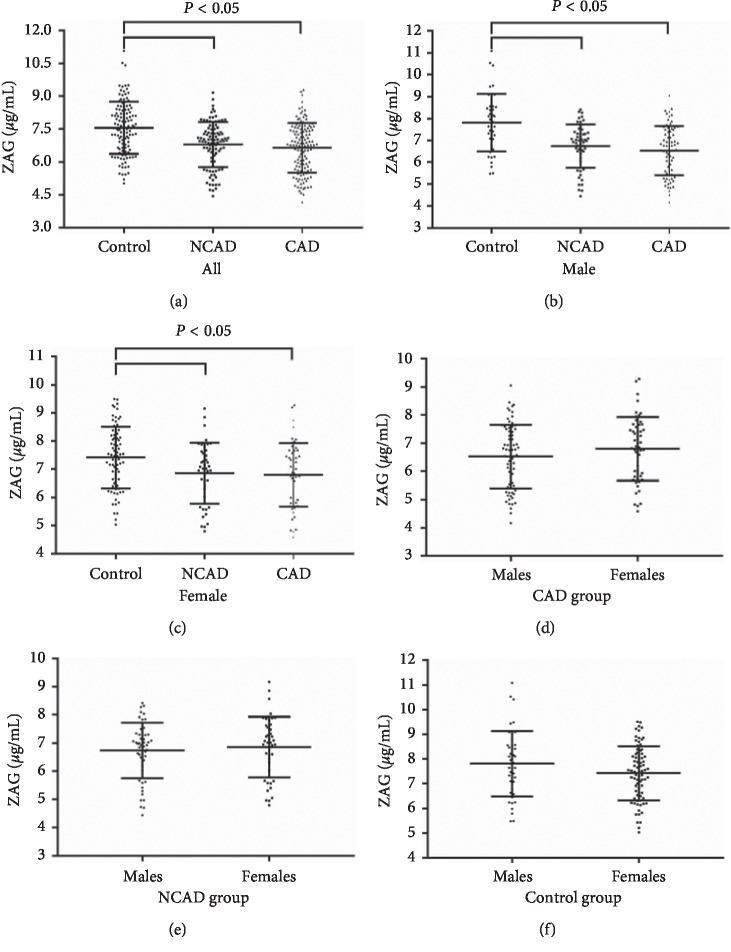
Serum ZAG levels in CAD, NCAD patients and controls. Serum ZAG levels in CAD, NCAD patients and controls of all subjects (a), males (b), and females (c), respectively. Serum ZAG levels of males and females in CAD (d), NCAD (e), and control (f) groups, respectively. CAD: coronary artery disease; NCAD: noncoronary artery disease; ZAG: zinc-*α*2-glycoprotein. All values are expressed as mean ± SD.

As shown in Figure 1(a), serum ZAG concentrations were significantly decreased in the CAD (6.64 ± 1.13 vs. 7.56 ± 1.19 *μ*g/mL) and NCAD groups (6.79 ± 1.03 vs. 7.56 ± 1.19 *μ*g/mL) compared to the control group (*P*<0.05). After further evaluation of serum ZAG levels in males (Figure 1(b)) and females (Figure 1(c)) separately, the significantly decreased serum ZAG levels in the CAD and NCAD groups still existed (6.53 ± 1.13 and 6.74 ± 0.99 vs. 7.81 ± 1.32 *μ*g/mL for males; 6.80 ± 1.12 and 6.86 ± 1.08 vs. 7.42 ± 1.09 *μ*g/mL for females, *P*<0.05). No significant sexual dimorphism was found in serum ZAG levels of the three groups as shown in Figures 1(d)-1(f).

### 3.3. Partial Correlations between Serum ZAG Levels and Clinical Parameters

Due to the mismatch in age and gender in the three groups, age-gender-adjusted Spearman's partial correlation analysis was used. As shown in Table 2, in all subjects, serum ZAG levels showed a positive association with urea (*r* = 0.122, *P* < 0.05). In the control group, serum ZAG levels were negatively correlated with DBP (*r* = −0.194), TC (*r* = −0.215), and Cr (*r* = −0.307) (all *P* < 0.05). In the CAD group, serum ZAG levels were positively associated with TC/HDL-C (*r* = 0.201), Cr (*r* = 0.270) and urea (*r* = 0.122) (all *P* < 0.05). No significant association was found between serum ZAG levels and other variables in the NCAD group ( all *p* > 0.05).

### 3.4. Stepwise Linear Regression Analysis for Variables Independently Related to Serum ZAG Levels in All Subjects

As presented in Table 3, the stepwise linear regression analysis showed that when serum ZAG was considered as the dependent variable with group (control, NCAD, and CAD), age, gender (male and female), BMI, SBP, DBP, FBG, TC, TG, HDL-C, LDL-C, ALT, Cr, and urea as independent variables, the variables including group (*β* = −0.328, *P* < 0.001) and urea (*β* = 0.106, *P*=0.046) were the independent contributors to serum ZAG levels. It is worth noting that the negative relationship between group and serum ZAG levels was consistent with the lower serum ZAG levels in CAD/NCAD patients as shown in Figure 1(a).

### 3.5. Multivariate Ordinary Logistic Regression Analysis for the Association of Serum ZAG Levels with the Risk of Increased Grouping

Table 4 displayed the results of the association of variables, including age, gender (male and female), BMI, SBP, DBP, FBG, TC, TG, HDL-C, LDL-C, ALT, Cr, urea, and serum ZAG levels (trisection: low, median, and high) with the risk of increased grouping by using the multivariate ordinary logistic regression analysis method. The results showed that age (odds ratio (OR) = 1.071, 95% confidence interval (CI) 1.045–1.099, *P* < 0.001), SBP (OR = 1.024, 95% CI 1.005–1.045, *P*=0.015), FBG (OR = 1.293, 95% CI 1.158–1.443, *P* < 0.001), and TC (OR = 5.960, 95% CI 1.820–19.511, *P*=0.003) were associated with the increased grouping levels, while TG (OR = 0.587, 95% CI 0.390–0.884, *P*=0.011), HDL-C (OR = 0.012, 95% CI 0.002–0.062, *P* < 0.001), and LDL-C (OR = 0.140, 95% CI 0.041–0.484, *P*=0.002) were associated with the decreased grouping levels. This result indicated that with the increased age, SBP, FBG, and TC and the decreased HDL-C, LDL-C, and TG, the risk of NCAD/CAD was increased with the increased grouping levels. Additionally, after grouping all subjects into three parts according to ZAG tertiles (lowest: <6.576 *μ*g/mL; median: 6.576–7.538 *μ*g/mL; and highest: >7.538 *μ*g/mL), we found that subjects in the lowest tertile of ZAG levels had 2.28-fold increased risk of grouping at one level higher when compared with those in the highest tertile levels (OR = 3.281, 95% CI 1.782–6.038, *P* < 0.001). This result indicated that the decreased serum ZAG levels were associated with the increased risks of NCAD/CAD.

### 3.6. Diagnostic Value of Serum ZAG Levels for CAD/NCAD Risks

**Figure 2:**
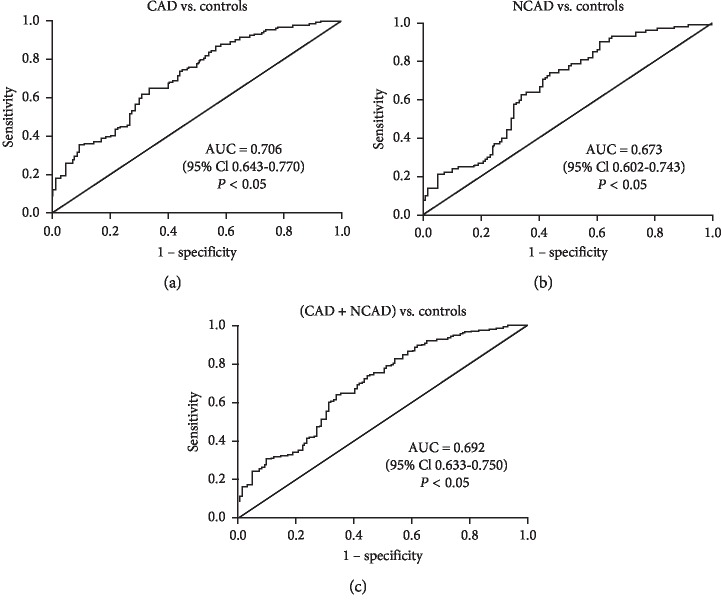
Comparison for ROC curves of serum ZAG in CAD/NCAD patients and controls. ROC curves were derived by plotting the relationship between the specificity and the sensitivity at various cutoff levels. ROC: receiver operating characteristic; AUC: area under the curve; CAD: coronary artery disease; NCAD: noncoronary artery disease; ZAG: zinc-*α*2-glycoprotein.

Finally, the diagnostic value of serum ZAG for CAD/NCAD was evaluated by ROC curves. As illustrated in Figure 2(a), ZAG could discriminate CAD patients from controls with an AUC of 0.706 (95% CI, 0.643–0.770, *P* < 0.05), a sensitivity of 65.12%, and a specificity of 66.12%. Additionally, as shown in Figure 2(b), ZAG could discriminate NCAD patients from controls with an AUC of 0.673 (95% CI, 0.602–0.743, *P* < 0.05), a sensitivity of 63.64%, and a specificity of 64.46%. Furthermore, as shown in Figure 2(c), ZAG could discriminate CAD and NCAD patients from controls with an AUC of 0.692 (95% CI, 0.633–0.750, *P* < 0.05), a sensitivity of 64.04% and a specificity of 66.12%.

## 4. Discussion

The main finding of our present study was that serum ZAG levels in patients with CAD and NCAD were significantly lower than those in controls. Grouping variable (control, NCAD, and CAD) was an independent determinant of serum ZAG levels, and ZAG levels were independently associated with the risk of grouping. The risk of grouping at one level higher in subjects with the lowest tertile of ZAG levels was 2.28-fold higher than those with the highest tertile levels.

ZAG was initially isolated from human plasma [31] and was subsequently proved to be a novel adipokine that can be secreted by adipose tissue and adipocytes [19]. Basic experiments have verified that ZAG could inhibit lipogenesis [21, 32], stimulate lipolysis [21, 32, 33] and *β*-oxidation [32, 33], promote white adipose tissue browning [33, 34], and thus play a critical role in regulating body weight. Recent clinical studies also found that serum ZAG levels were significantly lower in overweight/obese patients and were negatively correlated with BMI, waist circumstance, hip circumference, and fat mass [21, 22]. Beyond these observations, accumulating evidence revealed the close relationship between serum ZAG levels and various cardiometabolic risk factors, including cigarette smoking [26], dyslipidemia [27], hypertension [25], T2DM [23, 24], and metabolic syndrome [26–28]. These results indicated the potential role of ZAG in the development of cardiovascular diseases. In support of this hypothesis, previous studies performed by Smékal et al. in the Czech population [14] and our recently published paper in Chinese population [30] found the significant lower serum ZAG levels in PCAD patients (males <55y, females <65y) in comparison to the controls. Our present study further extended the available data in an elderly population and firstly found that serum ZAG levels were significantly lower in elderly CAD/NCAD patients from Chinese population.

Notably, our present study also showed that group (control, NCAD, and CAD) was an independent contributor to serum ZAG levels after controlling for other variables. After stratifying all subjects into trisections according to the serum ZAG tertiles, the risk of grouping at one level higher in subjects with the lowest tertile of ZAG was 2.28-fold higher than those with the highest tertile levels. That is to say, comparing with subjects with the high ZAG levels, subjects with the low ZAG levels have the higher risks of NCAD and CAD. As we knew, NCAD refers to individuals who cannot be diagnosed as CAD due to all coronary artery stenosis <50%, but already have abnormalities in glycolipid and cardiovascular metabolism. Interestingly, our recently published paper in PCAD patients also demonstrated that subjects with low tertile ZAG levels have higher probability of PCAD/NPCAD than those with high ZAG tertile levels [30]. Even after adjusting for other confounders, this phenomenon still existed [30]. In this respect, ZAG was indicated as a protective factor for CAD/NCAD, not only in the middle-aged population, but also in an elderly population, and ZAG may be a candidate biomarker of the diagnosis of CAD/NCAD.

Then, the diagnostic value of serum ZAG in distinguishing CAD/NCAD patients was further explored by ROC analysis. In our present study, we found that serum ZAG could distinguish CAD patients from controls with an AUC of 0.706, distinguish NCAD patients from controls with an AUC of 0.673, and distinguish NCAD and CAD patients from controls with an AUC of 0.692. The diagnostic of ZAG in CAD was supported by our previously published paper in a middle-aged Chinese population, where serum ZAG was found to be able to discriminate PCAD patients from controls with ROC curve area of 0.659 and 50.5% sensitivity and 78.0% specificity, respectively [30]. Similar conclusions were also drawn by Smékal et al. who performed the studies in the Czech individuals and also demonstrated that serum ZAG could serve as a diagnostic marker of PCAD with a ROC curve area of 0.894 and 73.3% sensitivity and 86.6% specificity [14]. All these findings together further confirmed the close relationship between serum ZAG levels and CAD, both in the middle-aged population and in an elderly population. However, the diagnostic value of ZAG for NCAD/CAD observed in our present study in an elderly population, of course, needed to be replicated in other populations, and a prospective cohort study also needed to be done for further evaluating the value of ZAG as a biomarker for CAD in the future.

Additionally, in recent years, there have been relatively few researches on the impact of gender on serum ZAG levels, and the results are controversial. In the present studies, no gender difference was found in serum ZAG levels and gender showed no significant association with serum ZAG levels in CAD patients. Inconsistent with our results, our recently studies in PCAD patients [30] and Selva et al. studies in simple obese patients [35] both found that gender was an independent factor contributing to serum ZAG concentrations in the multiple regression analysis. Yeung et al. performed studies in 258 Chinese populations randomly selected from the population-based Hong Kong Cardiovascular Risk Factor Prevalence Study and found that serum ZAG levels were higher in males than those in females [27]. Given the fact that the relationship between gender and serum ZAG is still controversial, more researches are still needed to be done in the future.

Finally, we found that urea was significantly higher in CAD patients in comparison with controls although Cr levels of them were in normal range. Moreover, urea was found to be independently positive associated with serum ZAG levels. In support of our results, Pelletier et al.'s study in patients with chronic kidney disease also found the positive relationship between serum ZAG and urea in multiple regression analyses [36]. In fact, a few past studies have suggested the close relationship between serum ZAG levels and renal function. Compared to the nonchronic kidney disease (CKD) controls, serum ZAG levels were almost 2.3-fold higher in patients with CKD stage 5 [36]. Our previous studies found that compared with those with the low tertile ZAG levels, T2DM patients with the high tertile ZAG levels were more likely to have mildly estimated glomerular filtration rate (eGFR) decrease [37]. Elhefnawy et al.'s study in T2DM showed that serum ZAG might serve as a potential biomarker for the early detection of diabetic nephropathy [37, 38]. *In vitro* studies performed by Schmitt et al. found that the addition of ZAG-enriched supernatant could significantly inhibit the proliferation of the primary proximal tubules epithelial cells, while knockdown of ZAG expression could increase the proliferation [39]. Further *in vivo* studies showed that ZAG was associated with the reduced epithelial proliferative reserve ability in the aged mouse kidneys [39]. As we mentioned above, subjects with the higher ZAG levels have the lower risks of CAD. That is to say, subjects with the higher ZAG levels are more protected for CAD, but perhaps more exposed to nephropathy. The relationship between serum ZAG and kidney functions in CAD patients and the possible mechanisms is still needed to be explored in the future studies.

Also, it is noteworthy to mention several limitations. First, our present study was carried out on a relatively small sample size and required further validation on larger subjects. Second, the generalizability of our findings might be limited because this was a single-center study performed in small samples of Chinese people. Third, although the present study investigated the association between serum ZAG levels and CAD, the causality of this association could not be explored due to the cross-sectional design.

## 5. Conclusions

Our present studies found that serum ZAG levels were significantly decreased in NCAD/CAD patients. The decreased serum ZAG levels were independently associated with the presence of NCAD/CAD. This study added to our previous studies in PCAD patients, showing that the decreased serum ZAG levels were associated with the increased risks of NCAD/CAD, not only in a middle-aged population, but also in an elderly population. ZAG might serve as a candidate diagnostic biomarker for NCAD/CAD. However, as this was a single-center study performed in small samples, further studies, especially in different races, are needed to better elucidate the diagnostic value of ZAG.

## Figures and Tables

**Table 1:** The demographic, clinical, and laboratory characteristics of CAD, NCAD subjects and controls.

Characteristics	Control group (*n* = 121)	NCAD group (*n* = 99)	CAD group (*n* = 129)
Age (years)	44.50 ± 12.97	60.12 ± 10.09^a^	61.03 ± 10.51^a^
Sex (M/F)	43/78	53/46^a^	75/54^a^
BMI (kg/m^2^)	26.44 ± 3.41	25.94 ± 3.74	25.88 ± 3.50
SBP (mmHg)	125.09 ± 18.70	129.82 ± 14.98	131.88 ± 18.35^a^
DBP (mmHg)	79.13 ± 11.77	76.46 ± 12.26	76.08 ± 12.03^a^
FBG (mmol/L)	5.36 ± 0.86	6.76 ± 2.48^a^	8.48 ± 3.77^ab^
TC (mmol/L)	4.81 ± 0.85	4.20 ± 0.97^a^	4.28 ± 1.09^a^
TG (mmol/L)	1.41 (0.87, 1.87)	1.28 (1.03, 1.87)	1.51 (1.07, 2.17)
HDL-C (mmol/L)	1.40 ± 0.36	1.08 ± 0.29^a^	1.02 ± 0.25^a^
LDL-C (mmol/L)	2.93 ± 0.70	2.42 ± 0.75^a^	2.55 ± 0.85^a^
TC/HDL-C	3.56 ± 0.93	4.02 ± 1.07^a^	4.34 ± 1.22^a^
LDL-C/HDL-C	2.20 ± 0.80	2.33 ± 0.76	2.61 ± 0.92^ab^
ALT (U/L)	19.00 (14.00, 25.00)	23.00 (16.00, 35.00)^a^	23.00 (17.00, 39.00)^a^
Cr (*μ*mol/L)	74.00 (58.00, 93.00)	74.00 (64.00, 84.00)	76.00 (65.00, 88.00)
Urea (mmol/L)	4.93 (4.00, 6.03)	5.15 (4.56, 6.58)	5.84 (4.81, 7.27)^a^
Hypertension (%)	0	65/99 (65.66)	94/129 (72.87)
Type 2 diabetes mellitus (%)	0	28/99 (28.28)	65/129 (50.39)^b^
Hyperlipidemia (%)	0	53/99 (53.54)	84/129 (65.12)
Hypotensive drugs (%)	0	56/99 (56.57)	79/129 (61.24)
Hypoglycemic drugs (%)	0	18/99 (18.18)	40/129 (31.01)^b^
Insulin (%)	0	5/99 (5.05)	11/129 (8.53)

M: male; F: female; BMI: body mass index; SBP: systolic blood pressure; DBP: diastolic blood pressure; FBG: fasting blood glucose; TC: total cholesterol; TG: triglycerides; HDL-C: high-density lipoprotein cholesterol; LDL-C: low-density lipoprotein cholesterol; ALT: alanine transaminase; Cr: creatinine; NCAD: noncoronary artery disease; CAD: coronary artery disease. Data were expressed as mean ± SD or median with interquartile range. ^*a*^*P* < 0.05 compared with the control group; ^*b*^*P* < 0.05 compared with the NCAD group.

**Table 2:** Partial correlation between serum ZAG levels and other variables.

Parameters	Serum ZAG levels			
	All (*r*) *n* = 349	Control (*r*) *n* = 121	NCAD (*r*) *n* = 99	CAD (*r*) *n* = 129
BMI (kg/m^2^)	−0.014	−0.073	0.024	0.052
SBP (mmHg)	0.056	−0.132	0.144	0.157
DBP (mmHg)	0.036	−**0.194**	0.165	0.007
FBG (mmol/L)	−0.084	−0.028	−0.197	0.124
TC (mmol/L)	0.082	−**0.215**	0.117	0.107
TG (mmol/L)	0.028	0.001	0.036	0.143
HDL-C (mmol/L)	0.038	−0.164	0.066	−0.107
LDL-C (mmol/L)	0.091	−0.134	0.081	0.094
TC/HDL-C	0.027	−0.043	0.055	**0.201**
LDL-C/HDL-C	0.042	−0.013	0.053	0.167
ALT (U/L)	−0.014	−0.094	0.077	0.093
Cr (*μ*mol/L)	0.043	−**0.307**	0.068	**0.270**
Urea (mmol/L)	**0.122**	0.048	0.065	**0.210**

BMI: body mass index; SBP: systolic blood pressure; DBP: diastolic blood pressure; FBG: fasting blood glucose; TC: total cholesterol; TG: triglycerides; HDL-C: high-density lipoprotein cholesterol; LDL-C: low-density lipoprotein cholesterol; ALT: alanine transaminase; Cr: creatinine; ZAG: zinc-a2-glycoprotein; NCAD: noncoronary artery disease; CAD: coronary artery disease. *r* represents age-sex-adjusted Spearman's partial correlation coefficients. Bold font means *P* < 0.05.

**Table 3:** Multiple stepwise regression analysis of independent factors associated with ZAG levels in all subjects.

Independent factors	Unstandardized coefficients (*B*) (95% CI)	Standardized coefficients (*β*)	*P* value
*Serum ZAG (R2* *=* *0.107)*			
Constant	7.804 (7.437–8.170)		0.037
Group (control, NCAD, CAD)	−0.474 (−0.625–0.324)	−0.328	<0.001
Urea	0.032 (0.001–0.064)	0.106	0.046

CI: confidence intervals. The following variables were also entered into multiple regression analysis but are not included in the equation: age; gender; BMI: body mass index; SBP: systolic blood pressure; DBP: diastolic blood pressure; FBG: fasting blood glucose; TC: total cholesterol; TG: triglycerides; HDL-C: high-density lipoprotein cholesterol; LDL-C: low-density lipoprotein cholesterol; ALT: alanine aminotransferase; Cr: creatinine.

**Table 4:** Ordinary logistic regression analysis of the risk of increased grouping in all study subjects.

Variables	Odds ratio (95% CI)	*P* value
Age	**1.071 (1.045–1.099)**	**<0.001**
BMI	1.012 (0.940–1.091)	0.749
SBP	**1.024 (1.005–1.045)**	**0.015**
DBP	0.973 (0.946–1.001)	0.060
FBG	**1.293 (1.158–1.443)**	**<0.001**
TC	**5.960 (1.820–19.511)**	**0.003**
TG	**0.587 (0.390–0.884)**	**0.011**
HDL-C	**0.012 (0.002–0.062)**	**<0.001**
LDL-C	**0.140 (0.041–0.484)**	**0.002**
ALT	1.010 (0.998–1.021)	0.095
Cr	0.996 (0.983–1.009)	0.542
Urea	1.006 (0.948–1.067)	0.842
*Gender*		
Male	1.00 (reference)	
Female	0.778 (0.434–1.395)	0.399
*Serum ZAG levels*		
Low	**3.281 (1.782–6.038)**	**<0.001**
Median	1.368 (0.736–2.540)	0.322
High	1.00 (reference)	

Multivariate ORs and 95% CIs from ordinary logistic regression models were used in the analysis. CI: confidence interval; BMI: body mass index; SBP: systolic blood pressure; DBP: diastolic blood pressure; FBG: fasting blood glucose; TC: total cholesterol; TG: triglycerides; HDL-C: high-density lipoprotein cholesterol; LDL-C: low-density lipoprotein cholesterol; ALT: alanine transaminase; Cr: creatinine; ZAG: zinc-a2-glycoprotein. Significant comparisons were in bold font.

## Data Availability

The datasets generated during and/or analyzed during the present study are available from the corresponding author upon reasonable request.

## Supplementary Materials

Supplementary Materials Serum ZAG levels were categorized into tertiles (lowest: ≤6.576* μ*g/mL; median: 6.576–7.538* μ*g/mL; highest: >7.538 *μ*g/mL) as presented in Table S1. The proportions of females in subjects with the median and the highest tertile ZAG levels were higher than those with the lowest tertile ZAG levels (*P* all < 0.05). Compared to subjects with the lowest tertile ZAG levels, Cr was significantly lower in those with the median ZAG levels (*P* < 0.05). Additionally, FBG was significantly lower while HDL-C was higher in subjects with the highest tertile ZAG levels in comparison with those with the median ZAG levels (*P* all < 0.05).
