# Supplementary material for: Serum Zinc-α2-Glycoprotein Levels in Patients with or without Coronary Artery Disease in Chinese North Population
Source: Int J Endocrinol. 2020 Feb 27;2020:7864721. doi: 10.1155/2020/7864721 (PMC7063206; doi:10.1155/2020/7864721)
Supplement: Supplementary Materials — Serum ZAG levels were categorized into tertiles (lowest: ≤6.576 μg/mL; median: 6.576–7.538 μg/mL; highest: >7.538 μg/mL) as presented in Table S1. The proportions of females in subjects with the median and the highest tertile ZAG levels were higher than those with the lowest tertile ZAG levels (P all < 0.05). Compared to subjects with the lowest tertile ZAG levels, Cr was significantly lower in those with the median ZAG levels (P < 0.05). Additionally, FBG was significantly lower while HDL-C was higher in subjects with the highest tertile ZAG levels in comparison with those with the median ZAG levels (P all < 0.05). [file 7864721.f1.docx]

Table S1 General characteristics of subjects with the low, middle, and high serum ZAG levels

| **Characteristics** | **Serum ZAG levels** | | |
| --- | --- | --- | --- |
|  | **Low** | **Median** | **High** |
| Age (years) | 56.31±13.09 | 55.68±13.18 | 53.11±14.58 |
| Sex (M/F) | 64/53 | 54/62 ^a^ | 53/63 ^a^ |
| BMI (kg/m^2^) | 25.87±3.17 | 25.94±2.98 | 26.47±4.32 |
| SBP (mmHg) | 128.83±17.35 | 128.44±17.35 | 129.56±18.76 |
| DBP (mmHg) | 77.27±12.31 | 77.15±11.42 | 77.33±12.51 |
| FBG (mmol/L) | 6.75 (5.23, 7.08) | 7.61 (5.20, 9.15) | 6.38 (4.90, 7.03) ^b^ |
| TC (mmol/L) | 4.40±1.13 | 4.34±0.98 | 4.58±0.90 |
| TG (mmol/L) | 1.74 (1.02, 1.99) | 1.59 (1.03, 1.88) | 2.02 (1.01, 2.26) |
| HDL-C (mmol/L) | 1.18±0.35 | 1.12±0.35 | 1.22±0.34 ^b^ |
| LDL-C (mmol/L) | 2.59±0.88 | 2.62±0.79 | 2.72±0.72 |
| TC/HDL-C | 3.94±1.21 | 4.07±1.05 | 3.96±1.13 |
| LDL-C/HDL-C | 2.34±0.87 | 2.48±0.83 | 2.35±0.86 |
| ALT (U/L) | 28.76 (17.00, 34.00) | 28.83 (16.00, 33.00) | 30.56 (14.00, 31.00) |
| Cr (μmol/L) | 83.16 (66.00, 89.50) | 74.02 (58.50, 85.00) ^a^ | 79.89 (64.00, 90.00) |
| Urea (mmol/L) | 5.96 (4.26, 6.29) | 5.55 (4.39, 6.34) | 6.54 (4.49, 6.72) |

Abbreviations: M: male; F: female; BMI: body mass index; SBP: systolic blood pressure; DBP: diastolic blood pressure; FBG: fasting blood glucose; TC: total cholesterol; TG: triglycerides; HDL-C: high-density lipoprotein cholesterol; LDL-C: low-density lipoprotein cholesterol; ALT: alanine transaminase; Cr: creatinine.

Data were expressed as mean ± SD or median with interquartile range.

^a^*P*<0 05 compared with the low ZAG levels group; ^b^*P*<0 05 compared with the median ZAG levels group.
